# NETosis as Source of Autoantigens in Rheumatoid Arthritis

**DOI:** 10.3389/fimmu.2016.00485

**Published:** 2016-11-14

**Authors:** Elisa Corsiero, Federico Pratesi, Edoardo Prediletto, Michele Bombardieri, Paola Migliorini

**Affiliations:** ^1^Centre for Experimental Medicine and Rheumatology, William Harvey Research Institute, Barts and The London School of Medicine and Dentistry, Queen Mary University of London, London, UK; ^2^Clinical Immunology and Allergy Unit, Department of Clinical and Experimental Medicine, University of Pisa, Pisa, Italy

**Keywords:** rheumatoid arthritis, neutrophils extracellular traps, histones, ectopic lymphoid structures, autoantigens, autoantibodies

## Abstract

In neutrophils (but also in eosinophils and in mast cells), different inflammatory stimuli induce histone deimination, chromatin decondensation, and NET formation. These web-like structures that trap and kill microbes contain DNA, cationic granule proteins, and antimicrobial peptides, but the most abundant proteins are core histones. Histones contained in NETs have been deiminated, and arginines are converted in citrullines. While deimination is a physiological process amplified in inflammatory conditions, only individuals carrying genetic predisposition to develop rheumatoid arthritis (RA) make antibodies to deiminated proteins. These antibodies, collectively identified as anti-citrullinated proteins/peptides antibodies (ACPA), react with different deiminated proteins and display partially overlapping specificities. In this paper, we will summarize current evidence supporting the role of NETosis as critical mechanism in the breach of tolerance to self-antigens and in supporting expansion and differentiation of autoreactive cells. In fact, several lines of evidence connect NETosis with RA: RA unstimulated synovial fluid neutrophils display enhanced NETosis; sera from RA patients with Felty’s syndrome bind deiminated H3 and NETs; a high number of RA sera bind deiminated H4 contained in NETs; human monoclonal antibodies generated from RA synovial B cells decorate NETs and bind deiminated histones. In RA, NETs represent on one side an important source of autoantigens bearing posttranslational modifications and fueling the production of ACPA. On the other side, NETs deliver signals that maintain an inflammatory milieu and contribute to the expansion and differentiation of ACPA-producing B cells.

## Introduction

NETosis was discovered as a new function of neutrophils and thoroughly investigated as an important mechanism in the protection against bacterial, fungal, and parasitic infections (1). When the size of microorganisms is excessive for phagocytosis (2), neutrophils activate an alternative pathway leading to the extrusion of decondensed chromatin fibers containing histones as well as antimicrobial granular and cytoplasmic proteins (3). NETs are released during a form of cell death, distinct from necrosis and apoptosis, which requires reactive oxygen species (ROS) produced by NADPH oxidase.

Recent data however challenge the prevalent view of NETosis as a cellular suicide. An early NETosis has been described, which occurs rapidly after exposure to microbial specific molecular patterns (e.g., within 60 min following *Staphylococcus aureus* stimulus), acts by a NADPH oxidase-independent pathway, and leads to the release of NET after nuclear envelope blebbing and vesicle formation, thereby preserving plasma membrane integrity. During this vital NETosis, cells are still able of some typical functions, such as chemotaxis and phagocytosis.

Slowly released from dying neutrophils or budding from live cells, NET fibers entrap microorganisms and represent a scaffold for enzymes, antimicrobial peptides, and ion chelators. These substances reach locally high concentrations and are thus able to cleave virulence factors and kill microorganisms (4).

Since the original description, it soon became apparent that both a defective and an excessive NET formation could have important consequences in human diseases, suggesting that a tight regulation of NETosis is critical to control pathogens while minimizing host damage.

When NET formation is impaired, as a result of NADPH oxidase or myeloperoxidase (MPO) deficiency (5), an immunodeficiency condition ensues, i.e., in chronic granulomatous disease, due to defective NADPH oxidase, restoration of NET formation by gene therapy allowed the control of severe fungal infection (6).

Conversely, a subset of neutrophils, identified for their lower density on gradients, is more abundantly represented in systemic lupus erythematosus (SLE) patients and is more prone to NETosis.

Netting neutrophils have not only been identified in nephritic kidneys in systemic lupus but also in ANCA-associated vasculitides (AAV), suggesting that NET constituents may be involved in the induction of severe manifestations of these systemic inflammatory disorders.

NET may also contribute to the pathogenesis of human diseases in a more subtle way, making potential autoantigens accessible to the immune system and creating the milieu where an autoimmune response may be triggered and fueled.

In this review, we shall summarize the current knowledge accumulated in recent years that point toward an important contribution of NET to the breach of immunological tolerance and the maintenance of autoimmunity and chronic inflammation in rheumatoid arthritis (RA).

## Neutrophils, Citrullination, and NETosis in RA

Neutrophils are the most abundant cells in the synovial fluid of RA patients although they appear a less important component of the chronic synovial inflammatory infiltrate where neutrophils are believed to only transiently populate the synovial tissue. In RA, circulating but especially tissue-infiltrating and synovial fluid neutrophils have all the features of activated cells, characterized by a prolonged survival and by the ability to secrete a wide range of inflammatory mediators including chemokines and cytokines (7). Neutrophil contribution to arthritis has been directly addressed in animal models such as antibody-induced arthritis (i.e., anti-collagen antibody-induced arthritis) or the transgenic KBxN mouse model. In these models, neutrophil depletion or interference with key signaling receptors (leukotriene B4 receptors, C5aR, CXCR1, and CXCR2) renders the mice resistant to disease induction. In RA, immune complexes engaging FcγRs activate neutrophils and trigger the release of ROS and proteases and the production of chemokines and cytokines. By means of these mediators, neutrophils recruit and modulate the function of other cell types, such as monocytes, dendritic cells, natural killer (NK), and lymphocytes, thus bridging innate and adaptive immunity (7).

A number of autoantibodies have so far been described in RA, but only anti-citrullinated proteins/peptides antibodies (ACPA) can be considered specific disease markers with sufficient specificity and sensitivity to be used as diagnostic tests (8). ACPA are a partially overlapping family of antibodies specific for protein sequences containing the aminoacid citrulline, the deiminated form of arginine residues (9, 10). ACPA display extensive genetic diversity and are characterized by somatic hypermutation in their variable Ig domains, suggestive of an antigen-driven response (11).

Indeed, it appears that the immune response to citrullinated epitopes is initially restricted but expands with time from the preclinical, immune phase of the disease to the clinical onset (12). Specifically, in the pre-disease stage of RA, the breach of immune tolerance to citrullinated antigens appear to be triggered in genetically predisposed individuals by protein citrullination at putative extra-articular sites, such as the periodontal tissue during *Porhyromonas gingivalis*-induced periodontitis or in the lung of smokers (13–15), which gives rise to a restricted ACPA repertoire. However, with the progression to clinical disease onset, epitope spreading and further affinity maturation of ACPA occurs (16).

In established RA patients, the targets of ACPA include autoantigens (i.e., filaggrin, fibrinogen, vimentin, collagen II, and histones) as well as exogenous antigens (i.e., alpha-enolase, EBNA-1, and EBNA-2 proteins). All these proteins become target of ACPA after deimination (or citrullination), a posttranslational modification (PTM) catalyzed by the calcium-dependent enzyme peptidyl arginine deiminase (PAD). PADs are inactive at intracellular calcium concentrations but can be activated by Ca2+ influx due to different stimuli: ionophore-induced macrophage apoptosis (extracellular calcium influx) (17) or lipopolysaccharide treatment of neutrophils (intracellular calcium mobilization). In neutrophils, PAD activation due to calcium influx takes place in the absence of caspase activation and triggering of apoptosis (18).

Furthermore, PADs can be released from the cell and become activated, as a result of the extracellular Ca2+ concentration (19).

Citrullination is a physiological process that regulates the homeostasis of several organs but is strongly amplified during inflammation. In RA, multiple proteins are citrullinated, especially in target organs of the disease, primarily the synovium but also in the lungs (20) and in myocardial tissue (21). In the RA joints, citrullinated fibrin is not only particularly abundant (22) but also several other citrullinated proteins including vimentin and aggrecan (23) are detectable. Citrullination is not an RA-specific process, and citrullinated proteins are present in several other inflammatory processes including the inflamed skeletal muscle tissue in myositis (24) and the synovium of spondyloarthritis patients (25). Currently, the existence of a citrullination profile typical of RA is still an unsettled issue.

Neutrophils contribute to protein citrullination in RA in several ways. Cells contained in synovial fluid (mainly neutrophils and monocytes) are characterized by the citrullination of a wide variety of proteins. Neutrophil exposed to a variety of stimuli (cytokines, TLR ligands) contain deiminated histones (18). Moreover, both perforin and complement membranolytic pathways lead to pore formation in the membranes, augmenting the intracellular calcium concentration and favoring the activity of PAD enzymes (26). Thus, granzyme B/perforin and complement activation with membrane attack complex (MAC) formation are able to induce in synovial fluid neutrophils an extensive protein citrullination.

**Figure 1:**
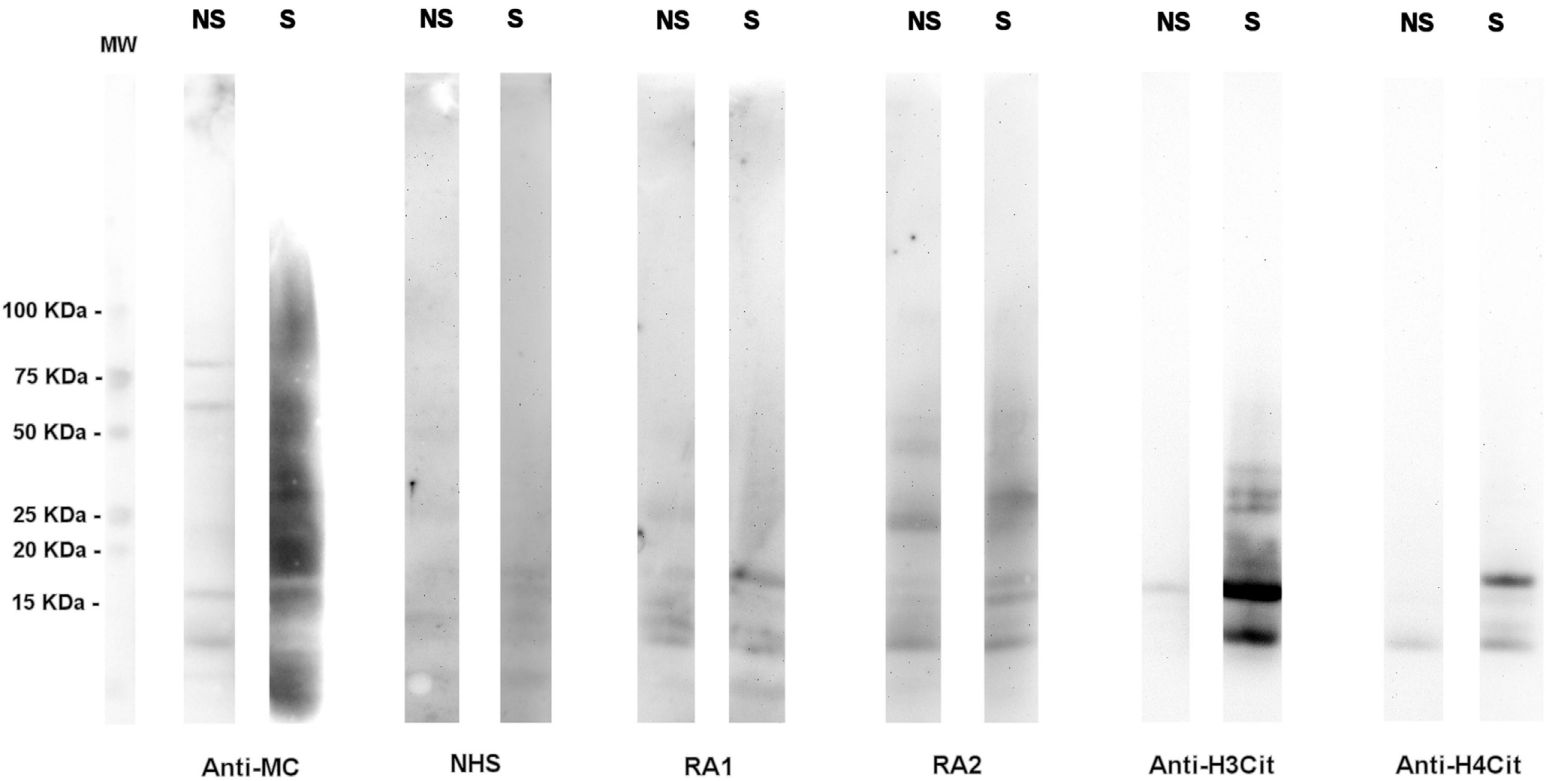
**A high number of citrullinated proteins is produced in neutrophils upon stimulation**. Total proteins from A23187-stimulated (S) or unstimulated (NS) neutrophils were subjected to SDS-PAGE and western blotting and probed with an anti-modified citrulline human monoclonal antibody (anti-MC), NHS or RA sera, anti-citrullinated H3 (anti-H3cit), or anti-citrullinated H4 (anti-H4cit) polyclonal antibodies. Anti-MC h-mAb decorates a high number of proteins in stimulated neutrophils, part of which are recognized by RA sera. Proteins of 10–15 KDa are bound by anti-MC h-mAb, by RA sera, and by anti-H4cit or anti-H3cit antisera, thus suggesting that in stimulated neutrophils, H3 and H4 are citrullinated and become target of RA immune response, as previously showed (57, with the permission of BMJ Publishing Group, N° 3976570562524).

Although a large spectrum of citrullinated proteins is produced by neutrophils in RA joints, the immune response detected in RA sera is relatively restricted (Figure 1).

A major contribution to the generation of citrullinated proteins comes from the propensity of RA neutrophils from peripheral blood or synovial fluid to form NET, either spontaneously or after LPS stimulation (27). Indirect evidence for the higher spontaneous NETosis of RA neutrophils comes from the observation of Dwivedi et al. who detected higher deiminated H3 content in RA as compared with controls (28). Moreover, exposure to RA immunoglobulins or purified ACPA induces NET formation, as already observed with autoantibodies of other specificities (see ANCA in AAV) (29), and netting neutrophils can be detected in synovial tissue and rheumatoid nodules from RA patients (27).

In synovial fluid, netting neutrophils release enzymatically active PAD2 and PAD4 that under the local conditions of inflamed joints may citrullinate extracellular proteins. Both soluble and NET-associated PAD can be detected, thus suggesting that NET may act as a molecular scaffold for protein citrullination (19).

Several recent works have evaluated the mechanisms behind NET regulation by PAD4. A single nucleotide polymorphism (SNP) at position 1858 (C1858T) in the DNA encoding a protein tyrosine phospatase (PTPN22), which results in the conversion of an arginine (R620) to a tryptophan (W620), has risen interest due to its strong connection with RA (30). Interestingly, Chang et al. (31) have investigated the correlation between C1858T and PAD4 to test the hypothesis that PTPN22 might negatively regulate protein citrullination independently from its phosphatase activity. They observed that PTPN22 is a strong inhibitor of PAD4 and that the presence of R620 is required for this inhibitory mechanism, which is lost in the presence of C1858T modification, thus resulting in an expansion of the pool of citrullinated antigens and possibly in an increase in NET formation. Despite their results, Chang et al. also highlighted that this SNP is not disease specific. Indeed, C1858T modification is present in other autoimmune diseases such as SLE or type 1 diabetes. Furthermore, only a subset of RA patients appears to carry this SNP.

NET from RA patients is also able to activate synoviocytes, upregulating the production of pro-inflammatory cytokines and amplifying joint inflammation.

Thus, neutrophils have an active role in the inflammatory process of RA not only regulating the function of other immune or structural cells (32) but also being the source of and posttranslationally modified autoantigens (33, 34).

An increased NETosis has also been observed in SLE. A peculiar subset of neutrophils, identified by density gradients as low density granulocytes (LDGs) and more frequently detectable in active SLE, has a pro-inflammatory phenotype (35) and forms spontaneously NET (36). On the other hand, it has been demonstrated that SLE patients have a decreased ability to degrade NET (37). Upon exposure to NET or NET components, activated caspase-1 is produced in macrophages, leading to the production of active IL-1β and IL-18 (38), while plasmocytoid dendritic cells are activated and release IFNalpha (39). Antimicrobial peptides like LL37 (40) and acetylated histones (41) are important mediators in these processes. Thus, NET represent on one side an important source of autoantigens fueling the production of anti-chromatin antibodies, on the other, a critical mechanism of disease induction, affecting several cell types and influencing the disease phenotype.

Increased NETosis has not only been described in type 1 diabetes, correlated with autoantibody titers and beta cell damage (42), but also in type 2 patients, and a direct role of hyperglycemia in increased NETosis has been shown (43).

More recently, an increased number of netting neutrophils has been reported in type 1 and type 2 diabetes, and their direct role in retarding wound healing has been demonstrated (44, 45).

## Histone Deimination in Neutrophils

The core nucleosome, comprising an H3–H4 tetramer and two H2A/H2B dimers is not a static DNA packaging structure, but on the contrary is a dynamic complex, and the modulation of its structure is an important component of transcriptional regulation.

Modifications in the conformation of histones, highly conserved in eukaryotic cells, from yeast to humans, are widely used in the dynamic modulation of chromatin structure and function. Indeed, evolutionary PTMs are more useful than amino acid substitutions.

So far, 20 types of histone PTMs have been described, which are able to modulate chromatin function by either altering the amino acid charge and consequently the inter-nucleosomal interactions or by enabling/inhibiting interactions with specific binding proteins external to nucleosomes but nonetheless essential for DNA regulation. Among all, one of the latest described is the deimination of arginine.

The first description of histone deimination was reported by the Yamada’s group (46). They observed that when HL60-derived granulocytes and peripheral blood granulocytes are stimulated with A23187 (a mobile ion-carrier known as calcium ionophore), their cytoplasmic PAD V deiminates histone H2A, H3, and H4 (other than nucleophosmin/B23). The percentage of deiminated histones detected in these studies was 10% of the total histone content.

In 2004, Cuthbert et al. (47) and Wang et al. (48) reported that PAD4 (correspondent to PAD V described by Yamada) deiminates histone H3 and H4 and has an impact on gene transcription by fine tuning the chromatin structure.

In particular, the group of Koutzarides (47) showed that PAD is activated when it is bound intracellularly by estrogen receptor. PAD deiminates histone H3 and H4 in different arginine located preferably in the N-terminal tail and increases the affinity of estrogen receptor for its target genes, thus resulting in a decrease of gene expression under the control of estrogen and thyroid hormones.

When PAD4 activity is inhibited by Cl-amidine, an increase in the expression of p53 and p53-related genes is observed as described by the group of Coonrod (48).

PAD4 is not the only PAD isoform involved in chromatin regulation. Indeed, Zhang et al. (49) suggested that stimulation of ERα-positive cells with 17β-estradiol (E2) promotes global citrullination of histone H3 arginine 26 (H3R26) on chromatin, catalyzed by PAD2 and not by PAD4, which instead deiminates H4R3.

Importantly, deimination may involve arginine but also methylarginine on H4 and H3 induced by PRMT1 and CARM1, respectively, thus dubbing PAD4 as a demethylating enzyme, thereby reverting the epigenetic modification of arginine methylation.

Moreover, deimination of the H2A/H2B dimer, probably involving three arginines (given the mass increase of 2.7 Da) stabilizes the dimer, making it less susceptible to harsh conditions than the native complex, as demonstrated by mass spectrometry analysis with increasing concentration of ammonium acetate (50).

Like core histones, extranucleosomal linker histones can also be the target of PAD activity. Christophorou et al. (51) recently demonstrated that H1 can also be citrullinated. In pluripotent stem cells, the presence of citrullinated H1 is highly correlated with the adoption of a more open state of chromatin and with a high level of transcription of pluripotency genes. Conversely, inhibition of PAD4 activity and consequently of H1 citrullination leads to a more compact state of chromatin and to higher transcription of differentiation genes. Dwivedi et al. demonstrated that H1 is an additional substrate for PAD4, providing evidence that during NETosis a variety of linker H1 can be deiminated on multiple arginines. Notably, H1.2 is deiminated on arginine 53, and the neo-formed epitope is thus recognized by specific anti-citrulline antibodies present in a small percentage of SLE and SS patients but not in RA (28).

Nevertheless the topic of histone deimination is still a tangled issue, given the high number of PTM co-expressed on histones and the limitations of the chemical and biological methods presently available for citrulline detection, which are not fully citrulline specific (52).

For instance, the antibody-detecting citrulline after chemical modification with antipyrine and 2,3-butanedione, the so called “Senshuo reagent,” also recognizes carbamylated proteins (53).

This lack of citrulline complete specificity is also a characteristic of some anti-unmodified citrulline antibodies commercially available.

To overcome this problem, recently Bicker et al. (54) suggested a rhodamine tagged phenylglyoxal derivative that can be used to directly visualize protein citrullination in a simple and highly sensitive quantitative method.

Besides technologies like mass spectrometry that allow determination of site-specific citrulline on proteins with high sensitivity but at high costs, the development of simple but nonetheless reliable and specific chemical or biological tools is a field still open to innovation.

Taken together, these results show that histone citrullination is a key regulatory mechanism for cell life, but in particular cells (neutrophils, eosinophils, mast cells, monocytes) histone deimination may lead to decondensation of the entire cell chromatin, thus affecting in an irreversible way the cell life and leading to ETosis (extracellular traps formation).

## Deiminated Histones and Autoantibodies in RA

Core histones are the most abundant proteins in NET (55), and several reports indicate that deiminated histones are a target of antibodies in RA. Specifically, sera from RA patients decorate NET, co-localizing on chromatin with anti-deiminated histone H3 antibodies. Moreover, sera from patients with Felty’s syndrome (characterized by RA, splenomegaly, and neutropenia) display a preferential binding to deiminated histones by ELISA, which was further identified to be directed against deiminated H3 using SDS-PAGE fractionated histones (56).

We have recently shown that RA sera, tested by immunoblot on acid-extracted proteins from calcium ionophore-stimulated neutrophils, frequently react with a band of 11 kDa. Its identity with deiminated H4 has been suggested by specific antibody recognition and demonstrated by MALDI/TOF analysis. The recognition of deiminated H4 has been confirmed by ELISA using either the entire molecule or citrullinated peptides corresponding to H4 sequences. When RA sera are tested with proteins contained in NET, the reactivity with a band identified as H4 is again detected. Moreover, by derivatization of citrulline residues, it has been shown that H4 contained in NET and recognized by RA sera is deiminated on arginine 23.

Antibodies specific for H4-derived citrullinated peptides (HCP1 – H4_14–34_ and HCP2 – H4_31–50_) are present in 67 and 63% of established RA (57). Their frequency is lower in early RA (37.3 and 48.5%, respectively), but they can be detected years before disease onset. As reported for other ACPA subtypes, anti-citrullinated histone antibodies precede symptom onset and predict disease development (58).

Similarly, antibodies against citrullinated sequences of H2A and H2B have been detected in healthy subjects that later develop RA. An increase in antibody frequency, together with the production of inflammatory cytokines, predicts the imminent development of clinically active RA (16).

Recently, citrullinated H2B has been detected as a target of autoantibodies in a high number of patients with established RA (59). RA synovial fluids contain high levels of citH2B and its immune complex, which have pro-inflammatory and immunostimulatory capacity.

Most importantly, Sohn et al. (59) demonstrated the arthritogenic potential of citH2B by immunization in a mouse model, although it was necessary to generate a low-grade articular inflammation to observe this peculiar effect.

On the whole, the definition of “true ACPA” and their pathogenic role in the initiation of arthritis is still a matter of debate. Production of citrulline-specific autoantibodies was non-detected in MRL-lpr/lpr and (NZB × B6)F1-hbcl-2-transgenic mice (60), and any arthritogenic role in Lewis and Brown-Norway rats was excluded (61). It has been later shown that immunization with citrullinated antigens like collagen II can enhance tissue injury and stimulate ACPA production in experimental arthritis (62, 63), and that administration of anti-citrullinated fibrinogen in collagen-induced arthritis enhances tissue injury (62). Genuine ACPA, which is ACPA non-reactive with the correspondent non-deiminated antigen, are actually detectable in mice, but the production of these antibodies is highly dependent on the mouse strain, the antigen used for immunization and ACPA detection, and the immunization protocol (64). Further support for a pathogenic role of the immune response to citrullinated antigens derives from a recent report on the immunomodulatory potential of synthetic citrullinated antigens. In rats, a tolerogenic injection protocol using synthetic multiepitopes derived from common citrullinated proteins ameliorates adjuvant-induced arthritis, inducing an expansion of regulatory T cells and a reduction of Th17 cells (65).

## Ectopic Lymphoid Structures as a Source of Anti-Net Antibodies in RA

Approximately 50% of patients with RA are characterized by the presence of clusters of infiltrating lymphomononuclear cells in the RA joint synovium forming ectopic lymphoid structures (ELS). Synovial ELS not only resemble secondary lymphoid organs (SLO) but they can also support a germinal center (GC) response. In particular, ELS are characterized by segregation of T and B lymphocytes, differentiation of high endothelial venules (HEVs), and networks of stromal follicular dendritic cells (FDC) (66, 67). Moreover, ELS are functional structures supporting the affinity maturation and clonal selection of autoreactive B cells bringing to the differentiation of plasma cells producing antibodies toward citrullinated antigens (68). Thus, ELS in the RA synovium can directly sustain autoimmunity by not excluding self-reactive B cells to affinity mature into the ectopic GC. This process is also antigen and disease specific. In RA, ectopic GC support the production of antibodies against citrullinated proteins (68–70), in other autoimmune diseases, they can support the production of antibodies targeting other autoantigens, i.e., in Sjögren’s syndrome ribonucleoprotein Ro/La (71, 72), in Hashimoto’s thyroiditis thyroglobulin and thyroperoxidase (73), and in myasthenia gravis acetylcholine receptor (74).

Of relevance, we have recently demonstrated that up to 40% of recombinant monoclonal antibodies derived from single CD19+ synovial tissue cells (RA-syn-rmAbs) obtained from ACPA+ RA patients with functional ectopic GC display reactivity toward citrullinated histones (75). In particular, in this work we not only showed a strong reactivity of the RA-syn-rmAbs toward citrullinated histone H2A and H2B but also citrullinated vimentin and fibrinogen. Importantly, the reactivity against histones was confirmed in a cell-based NETs co-localization assay using either RA synovial fluid or circulating neutrophils as cell substrate. These antibodies were thus defined as anti-NETs antibodies. Moreover, the anti-NETs immunoreactivity was shown to be acquired within the synovial microenvironment in the ectopic GC through affinity maturation and intra-synovial diversification and was lost when the Ig H and L variable regions were reverted to their germline sequences (75).

As discussed above, NETs formation is critically dependent on histone citrullination, and citrullinated histones comprise around 70% of all NETs proteins. A proteomic analysis of NETs derived from healthy control neutrophils has identified at least 25 different proteins that decorate these chromatin structures, such as citrullinated vimentin and α-enolase, which are also targets of ACPA, as discussed above (27). Therefore, a delay in the clearance of NET could form a reservoir of citrullinated and non-citrullinated antigens in the extracellular space of the RA joint and may contribute to the autoimmune response in RA. Indeed, defects in NET clearance have been already associated with other autoimmune diseases like lupus nephritis. In particular, defects in DNase1, which is responsible to degrade NET, have been observed in SLE patients (37).

As discussed in the Section “Introduction,” although the local release of NET in the RA joint represents an important source of citrullinated autoantigens, additional sources of NET-related autoantigens should also be considered during the generation of ACPA, particularly in the preclinical immune phase of RA. Environmental factors such as bacterial infection during periodontal disease (i.e., *P. gingivalis*) (76, 77) and smoking (78) can bring to the formation of citrullinated antigens outside the joint and can lead to ACPA production before the clinical onset of the disease. Of interest, *P. gingivalis* is the only known pathogen expressing PAD, which can citrullinate both the endogenous and host proteins, thus supporting ACPA formation (79, 80).

However, periodontitis is also characterized by an increased production of antibodies toward the non-citrullinated form of RA-associated antigens, suggesting that in some patients periodontal disease could break the tolerance to non-modified autoantigens in the preclinical phase of RA (81).

In periodontal disease (82, 83), NETosis is increased, and NETs are also involved in acute and chronic lung inflammation (84). In the subset of ACPA-positive RA patients, lung abnormalities are detectable by HCRT early in the disease course, associated with the presence of citrullinated proteins in the lungs and ACPA in the bronchoalveolar lavage fluid (85). The level of ACPA and the production of ACPA of different specificity are both predictive of the development of interstitial lung disease (86). Bronchial biopsies from early RA patients show an inflammatory infiltrate containing T cells, B cells, and plasma cells and occasionally GC-like structures (87). In early RA, synovia, periodontal tissue, or the lung share an increased content of citrullinated proteins, evidence of NETosis, and presence of ectopic GCs. It is conceivable that elevated concentration of NET-associated proteins due to an increase in NETs production or NETs removal impairment could locally provide a source of citrullinated autoantigens able to trigger an autoimmune response.

NETs expose citrullinated proteins together with danger signals like cathelicidin or HMGB1 that promote the activation and maturation of professional antigen-presenting cells. NETs uptake and processing may allow the expansion of T cells able to support the affinity maturation and clonal diversification of B cells, leading to ACPA production.

**Figure 2:**
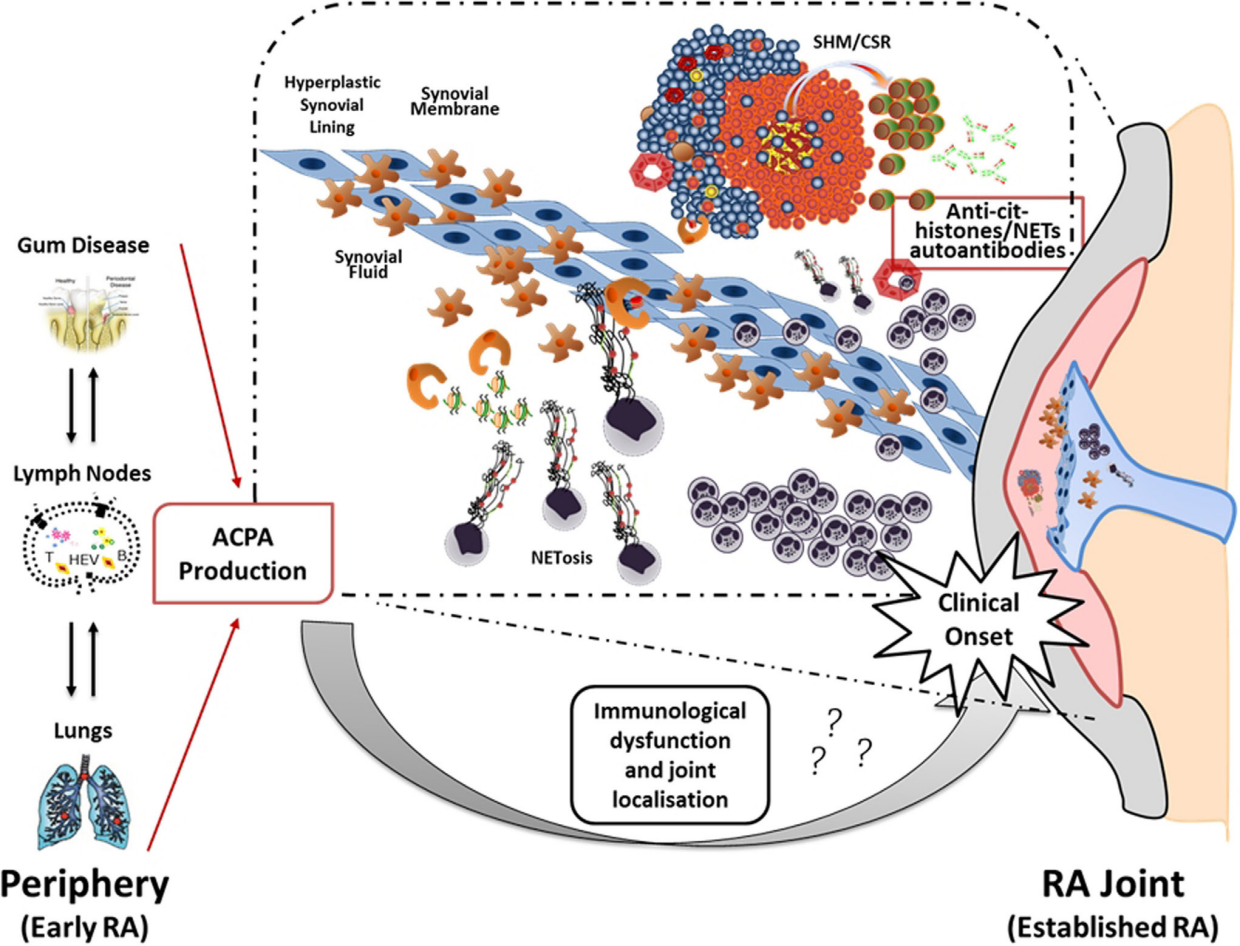
**Neutrophil NETosis and anti-NET antibodies in synovial ELS: a new model suggesting linking inflammation and autoimmunity to citrullinated proteins in RA**. The figure illustrates the hypothetical evolution of ACPA from a pre-disease phase outside the joint to a disease phase associated with chronic synovial inflammation. In particular, it has been proposed that environmental factors such as bacterial infection in periodontal disease and smoking can lead to the formation of citrullinated antigens outside the joint which, in the context of HLA-DRB1 shared epitope, can lead to the production of ACPA prior to the clinical onset. However, the second inflammatory hit that occurs in the synovium is not clear yet. Here, we proposed that migration of neutrophils from the periphery followed by aberrant neutrophil NETosis during chronic inflammation within the RA compartment (synovial tissue and fluid) provide a continuous source of externalized citrullinated antigens, such as citH2A, citH2B, and citH4 histones that can be presented by antigen-presenting cells following engulfment of neutrophils nuclear fragments. Such process would sustain an antigen-driven autoimmune response toward citrullinated antigens within ELS in the RA joint resulting in the production of anti-NET autoantibodies, which may contribute to the perpetuation of chronic inflammation and autoimmunity (75).

Thus, the current hypothesis is that the local release of citrullinated histones and probably other citrullinated and non-citrullinated antigens during an excessive NETosis can sustain the antigen-driven generation of high affinity anti-NETs antibodies within ELS with functional ectopic GC (Figure 2).

## Author Contributions

All authors listed have made substantial, direct, and intellectual contribution to the work and approved it for publication.

## Conflict of Interest Statement

The authors declare that the research was conducted in the absence of any commercial or financial relationships that could be construed as a potential conflict of interest.
